# A retrospective descriptive analysis of rabies post-exposure prophylaxis cases in Dalian from 2016 to 2017

**DOI:** 10.1017/S0950268819000785

**Published:** 2019-05-17

**Authors:** Yi Song, Xiaoguang Lu, Xue Jiang, Weitong Zhao, Zhiwei Fan, Xin Kang, Yu Wang

**Affiliations:** 1 Emergency Department, Affiliated Zhongshan Hospital of Dalian University, Dalian 116001, China; 2 Graduate School, Dalian University, Dalian 116622, China; 3 Graduate School, Zunyi Medical University, Zunyi 563003, China

**Keywords:** Economic burden, post-exposure prophylaxis, rabies, vaccine

## Abstract

Dalian, China, is a city free of rabies in recent 20 years, but the annual cost for rabies vaccination still brings an economic burden on society and individuals. We did a retrospective descriptive analysis to analyse the reason for this and try to find some ways to resolve it. A total of 10 028 post-exposure prophylaxis (PEP) cases were recorded from January 2016 to December 2017. According to the exposure grades, 32 cases were grade I; 7712 cases were grade II; 2284 cases were grade III. All the patients in the cases were injured by pet dogs without abnormal clinical signs, and 80% of them were home pet dogs. Fifty-two per cent of the pet dogs were vaccinated. All the dogs survived during the PEP vaccination period. The data showed that a considerable proportion of people who did not have exposure risk for rabies had received vaccination. The underlying reasons included social, medical and personal factors. So here we proposed to replace the current ‘five-course’ intramuscular injection with intradermal injection method in the cities free of rabies in China, this can not only achieve effective vaccination but also save resources and eliminate the fear of rabies from victims. Meanwhile we should strengthen communication on rabies knowledge and make a routine evaluation of rabies surveillance system to improve understanding of the risk for rabies from biting animals.

Although China is still an endemic country of rabies, the incidence of human rabies has been geographically limited in recent years, which is high in the south but low in the north, and even no human case has occurred in some northern provinces in the past decade [1, 2]. Dalian is a coastal city located in the north of China. Our institution is a tertiary municipal hospital affiliated with Dalian University responsible for the administration of rabies vaccines in the area of Zhongshan District (one of the four districts in Dalian), and there are about 5000 patients bit by dogs visiting our hospital for rabies vaccination each year. We adopt the ‘five-course’ vaccination programme by intramuscular injection and not regularly give human rabies immunoglobulin except for severe grade III exposure in our institution. The cost per patient is about $58, which is about $ 290 000 annual, and the indirect costs have not been included. The whole cost for rabies vaccination brings an economic burden on society and individuals. In light of this, we analysed the rabies post-exposure prophylaxis (PEP) cases to our hospital from 2016 to 2017, to find out whether there were some unnecessary vaccination cases. By investigating the underlying reasons, we wanted to provide some ideas for solving these problems.

A retrospective descriptive analysis of the clinical data on rabies vaccination from the emergency department at Zhongshan Hospital, Dalian University was conducted using frequencies and percentages for categorical variables. The clinical data consisted of dog ownership categories (1. own home pet dog; 2. other home pet dog; 3. stray dog), exposure grades (grade I: touching or feeding an animal or licks on intact skin; grade II: nibbling of uncovered skin, minor scratches or abrasions without bleeding; grade III: single or multiple transdermal bites or scratches, contamination of mucous membranes with saliva from licks, licks on broken skin) [3], dog healthy status (1. normal; 2. mad or suspected mad; 3. uncertain), dog vaccination (vaccinated explicitly within 1 year before the time of dog bit: 1. yes; 2. no) and dog living status after 28 days (1. as usual; 2. death; 3. no trace). The data were collected from January 2016 to December 2017. Percentage of dogs vaccinated, own home pet dog, exposure to grade II and grade III were, respectively, calculated and delineated with the month. The statistical analysis was performed using the SPSS (Version 20.0). Correlations between the number of vaccinated cases and the proportion of the dog population vaccinated, own home pet dog, exposure to grade II and grade III were assessed by Spearman correlation. *P* < 0.05 was considered statistically significant.

From January 2016 to December 2017, a total of 10 028 patients came to our hospital for rabies PEP. According to the exposure grades, 32 cases were grade I (0.3%), 7712 cases were grade II (76.9%), 2284 cases were grade III (22.8%), the percentage of each grade was relatively stable and did not change much with the month (Fig. 1). All the patients in the cases were injured by pet dogs without abnormal clinical signs, and 80% of them were home pet dogs. But only 52% of the pet dogs were vaccinated which was not high, and the percentage of dogs vaccinated fluctuated greatly with the month (Fig. 1). All the dogs survived during 28 days of observation (the duration of five-course vaccination). The detailed data were shown in Table 1. Correlation analysis showed a positive correlation between the number of vaccinated cases and own home pet dog (*r* = 0.991, *P* < 0.05), exposure grade II (*r* = 0.880, *P* < 0.05), exposure grade III (*r* = 0.644, *P* < 0.05). Correlation analysis showed no statistical significance between the number of vaccinated cases and the proportion of the dog population vaccinated (*r* = −0.001, *P* > 0.05). Vaccination coverage amongst dogs did not associate with human dog bite cases in our study.

**Fig. 1.**
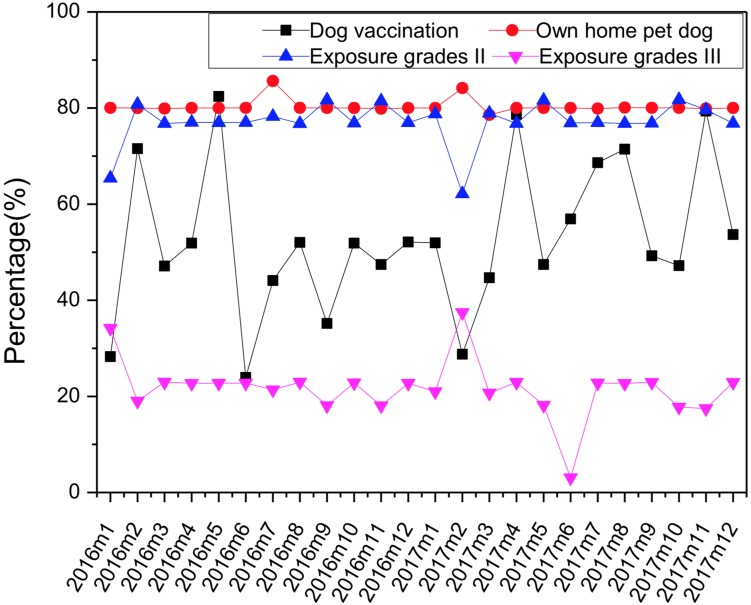
Percentage of dogs vaccinated, own home pet dog, exposure to grade II and grade III change with the month (January 2016–December 2017).

**Table 1.** The clinical data of rabies vaccination cases

Date	Cases (No.)	Dog ownership category (No.)			Exposure grades (No.)			Dog healthy status (No.)			Dog vaccination (No.)		Dog living status 28d (No.)		
		Own home pet dog	Other home pet dog	Stray dog	I	II	III	Normal	Mad or suspected mad	Uncertain	Yes	No	As usual	Death	No trace
Jan. 2016	506	405	101	0	2	331	173	506	0	0	143	363	506	0	0
Feb. 2016	510	408	102	0	1	412	97	510	0	0	365	145	510	0	0
Mar. 2016	418	334	84	0	1	321	96	418	0	0	197	221	418	0	0
Apr. 2016	370	296	74	0	1	285	84	370	0	0	192	178	370	0	0
May. 2016	330	264	66	0	1	254	75	330	0	0	272	58	330	0	0
Jun. 2016	426	341	85	0	1	328	97	426	0	0	102	324	426	0	0
Jul. 2016	501	429	72	0	2	392	107	501	0	0	221	280	501	0	0
Aug. 2016	396	317	79	0	1	304	91	396	0	0	206	190	396	0	0
Sept. 2016	415	332	83	0	1	339	75	415	0	0	146	269	415	0	0
Oct. 2016	320	256	64	0	1	246	73	320	0	0	166	154	320	0	0
Nov. 2016	432	345	87	0	2	352	78	432	0	0	205	227	432	0	0
Dec. 2016	330	264	66	0	1	254	75	330	0	0	172	158	330	0	0
Jan. 2017	410	328	82	0	1	323	86	410	0	0	213	197	410	0	0
Feb. 2017	518	436	82	0	2	322	194	518	0	0	149	369	518	0	0
Mar. 2017	546	429	117	0	2	431	113	546	0	0	244	302	546	0	0
Apr. 2017	375	300	75	0	1	288	86	375	0	0	295	80	375	0	0
May. 2017	430	344	86	0	1	351	78	430	0	0	204	226	430	0	0
Jun. 2017	390	312	78	0	2	300	12	390	0	0	222	168	390	0	0
Jul. 2017	408	326	82	0	1	314	93	408	0	0	280	128	408	0	0
Aug. 2017	392	314	78	0	2	301	89	392	0	0	280	112	392	0	0
Sept. 2017	406	325	81	0	1	312	93	406	0	0	200	206	406	0	0
Oct. 2017	411	329	82	0	2	336	73	411	0	0	194	217	411	0	0
Nov. 2017	378	302	76	0	1	301	66	378	0	0	300	78	378	0	0
Dec. 2017	410	328	82	0	1	315	94	410	0	0	220	190	410	0	0
Total	10 028	8064 (80%)	1964 (20%)	0	32 (0.3%)	7712 (76.9%)	2284 (22.8%)	10 028	0	0	5188 (52%)	4840 (48%)	10 028	0	0

Whether people need to be vaccinated against rabies after being bit by dogs depends fundamentally on whether they are exposed to rabies virus (RABV). WHO concludes that determination of whether exposure to RABV has occurred should include consideration of factors such as the epidemiology of rabies in the country; the severity of exposure; the species and clinical features of the animal; the vaccination status of the animal; the animal's availability for observation; and the results of laboratory testing [3].

According to WHO, a country or area is defined as free of dog rabies if no indigenously acquired dog-mediated rabies cases have been confirmed in humans, dogs or any other animal species for at least 2 years [3]. In the recent 20 years, there are no cases of human [4] or animal rabies according to the local Animal Epidemic Disease Control and Prevention Center in Dalian, so it can be regarded as a ‘non-rabies epidemic area’. Referring to the above non-endemic status, most of the rabies vaccine-immunised population in our hospital can exclude the possibility of exposure to rabies in the past 2 years. If we followed the WHO rabies PEP recommendations, only 4840 cases (dogs not vaccinated) in our study should have been vaccinated and no one should have received the five-course vaccine because all the dogs lived more than 10 days [3, 5], which would save about $410 000. Maybe the number of persons receiving PEP could be reduced if the money saved by this was used to encourage greater vaccination of dogs. We think it is the result of comprehensive factors that make these people still come for vaccination. The first is the fear of rabies that is embedded in the residents. At present, most Chinese people come to know rabies mainly through the Internet, media or rumours of the crowd. Such knowledge is partial, that it is generally known that the mortality rate of rabies is 100%, but little is known about its pathogenesis [6]. Therefore, victims are under tremendous psychological pressure, they cannot get rid of the fear of rabies even if they are clearly injured by vaccinated and healthy pet dogs. Even a few people suffer from lyssophobia [7], at this moment the rabies vaccine is not only a prophylactic immune agent but also a psychological ‘placebo’. The second important factor is the potential ‘dispute’ that medical institutions may face. For medical institutions, lacking experienced veterinarian and objective support of fast and effective test for rabies virus, it is hard for the clinicians to judge whether an animal is healthy or not by its clinical manifestations. Being lack of relevant policy protection, the medical institutions and clinicians might get sued or punished once a rabies case occurs even they follow the WHO rabies PEP recommendations. So at the annual meeting of rabies in China held by the Chinese Preventive Medicine Association, the leading experts have strongly suggested that people bit by dogs should receive the full PEP course under any condition. The China Center for Disease Control and Prevention has organised the experts to develop the technical guideline for human rabies prevention and control following the WHO recommendations in 2016 [8]; however, its effective implementation urgently needs the health administration to issue relevant laws to protect medical institutions and clinicians operating in accordance with this guideline. Above all, the medical institutions provide PEP to cases of dog bites regardless of any of the determinants suggested by the WHO.

WHO promotes the use of intradermal administration of rabies vaccines, which can result in an equivalent immune response at a lower dose, thereby reducing the volume used and the direct cost of vaccine by 60–80% in comparison with standard intramuscular injection [9]. It has been successfully introduced to many countries throughout Asia and Africa. So we can initially promote intradermal injection in the cities free of rabies in China. This measure can not only achieve effective vaccination, but also save resources and eliminate the fear of rabies from victims. Meanwhile, the government should strengthen communication and education on rabies knowledge. It is also important for us to make a routine evaluation of rabies surveillance system to improve the understanding of the risk for rabies from biting animals. These together can increase adherence to vaccination recommendations and reduce unnecessary administration of vaccine.

The launch of the Global Rabies Framework in 2015 celebrated the proof of concept that rabies can be eliminated in various settings and the shared goal of reaching zero human deaths from rabies by 2030, worldwide. But in China, it will take many generations to eliminate the fear for rabies which has become so firmly ingrained in the minds of people.
